# Improving Upconversion Efficiency Based on Cross-Patterned Upconversion Material Slot Waveguides on a Silicon Layer

**DOI:** 10.3390/nano9040520

**Published:** 2019-04-03

**Authors:** Youngsoo Kim, Kihwan Moon, Young Jin Lee, Seokhyeon Hong, Soon-Hong Kwon

**Affiliations:** Department of Physics, Chung-Ang University, Seoul 06974, Korea; youngsoo.kim94@gmail.com (Y.K.); sinbadra@gmail.com (K.M.); youngjin.lee.91@gmail.com (Y.J.L.); lechter@naver.com (S.H.)

**Keywords:** upconversion, wavelength conversion, energy harvesting, slot mode, Purcell effect

## Abstract

Upconversion (UC) materials can be used to harvest near-infrared (NIR) light and convert it into visible light. Although this improves optical device operating spectral range and efficiency, e.g., solar cells, typical UC material conversion efficiency is too low for practical devices. We propose a cross-patterned slot waveguide constructed from UC material embedded in a high index semiconductor layer to improve UC. Since the slot waveguide mode is induced in the low index UC slot, NIR absorption (~970 nm) increased 25-fold compared with film structures. Furthermore, the spontaneous emission enhancement rate at 660 nm increased 9.6-fold compared to the reference film due to resonance excited in the UC slot (Purcell effect). Thus, the proposed UC slot array structure improved UC efficiency 240-fold considering absorption and emission enhancements. This double resonance UC improvement can be applied to practical optical devices.

## 1. Introduction

Upconversion (UC) materials convert lower energy light, which cannot be used in optical devices due to active material spectral response limitations, to higher energy light that can be effectively captured. Hence, they have been studied in many fields, including bio-imaging [1,2,3] and energy harvesting [4,5,6,7,8,9]. However, UC material absorption and emission rates are not high enough for practical uses in optical devices [10]. Although many studies have proposed improving UC efficiency by controlling material composition or doping [11,12], efficiency remains insufficient for practical devices. On the other hand, strong optical fields induced in nanophotonic structures have been shown to improve UC efficiency [13,14]. Strong electric fields at incident wavelength increase light–matter interaction in UC materials, enhancing absorption efficiency. Strong electric fields at emission wavelengths can also enhance the emission rate through the Purcell effect [15,16]. Significant UC efficiency improvements have been demonstrated for plasmon resonances in metallic structures [7,17] and Mie resonances in UC material dielectric rods [18,19,20,21].

In particular, more sophisticated UC layer design rules are required for effective UC material application in solar cells. The conventional solar cell active layer absorbs visible light and should be retained with minimum modification to retain the original efficiency with respect to visible light. However, solar cell UC material layers should provide high conversion efficiency from near-infrared (NIR) to visible light [4,5,22,23].

Therefore, we propose a cross-patterned UC slot waveguide structure on a solar cell with a high refractive index semiconductor active layer, e.g., silicon. This structure maintained the original active layer visible light absorption, since the UC material had a small area filling factor (0.0975). However, the introduced UC slot waveguide structure will significantly enhance the conversion efficiency due to the strong electric field in the low refractive index slot waveguide. In particular, the proposed structure strongly enhanced NIR (980 nm) absorption and visible emission (660 nm) from coupling with slot waveguide modes.

## 2. Upconversion Material Absorption Enhancement

In this paper, we used a home-made three-dimensional (3D) finite-difference time-domain (FDTD) method for simulations of the proposed structure. Spatial resolution of 5 nm was used both for the absorption and emission simulations. In the simulations of the absorption, periodic boundary conditions with a square unit cell were assumed for *X* and *Y* axes to represent the proposed periodic structure, and the perfectly matched layer (PML) was on the *Z* axis to indicate infinite free space. The incident light was represented by a planewave monochromatic source with changing the wavelength. In the simulations of the emission, PML boundary conditions were applied to *X*, *Y*, and *Z* axis to represent infinite space and the horizontal simulation domain was 10 × 10 square lattices of the structure to avoid the periodic interference effect of a dipole source. All light from a dipole source can escape into free space less than 10 lattice period. A linearly-polarized dipole source with an emission wavelength was placed in the region of the UC slot. For the calculations of total output, the Poynting vector was integrated over the surface of the rectangular box including the proposed structure, where the surface was separated 100 nm away from the structure.

**Figure 1:**
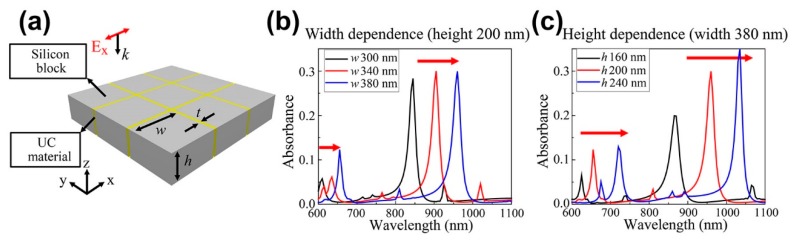
(**a**) Proposed cross-patterned upconversion (UC) material slot waveguide structure on silicon thin layer, where *w* and *h* are the width and height of each silicon layer block, respectively, and *t* is the slot thickness; simulated Er^3+^/Yb^3+^ doped NaYF_4_ UC material slot absorbance spectra for different (**b**) widths and (**c**) heights.

Figure 1a shows the proposed cross-patterned UC slot waveguides in a thin silicon layer to enhance UC material conversion efficiency. The UC slot waveguide array forms a square lattice to provide polarization-independent absorption for incident light. Figure 1b,c shows two strong light absorption peaks when Ex polarized incident light propagates into the structure, e.g., structure with *w* = 380 nm, *h* = 200 nm, and *t* = 20 nm exhibits peaks at 660 nm and 970 nm (P1 and P2 respectively). Hence, the proposed UC slot waveguide has two resonance modes. The lattice design ensures differently polarized incident light, e.g., *Ey* polarization, is maintained.

Resonant wavelengths can be controlled by changing the silicon block width or height as shown in Figure 1b,c, respectively. For *w* = 300 to 380 nm at fixed *h* = 200 nm, P1 and P2 redshift from 620–660 nm and 850–970 nm, respectively; whereas for *h* = 160 to 240 nm at fixed *w* = 380 nm, P1 and P2 increase 630–730 nm and 880–1040 nm, respectively.

**Figure 2:**
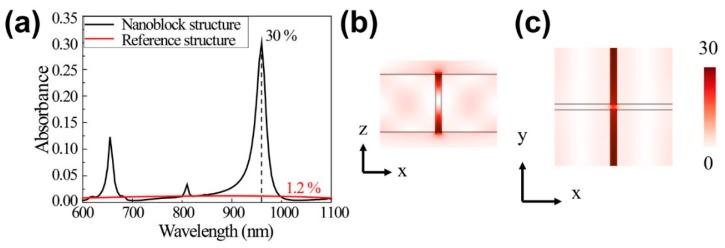
(**a**) Simulated absorption spectra for the proposed upconversion (UC) slot structure and reference film; the UC device exhibits emission and absorption resonances at 660 and 970 nm, respectively; (**b**) and (**c**) electric field intensity profiles for 970 nm incident light; maximum field intensity is almost 30 times that of the incident light.

Figure 1 shows that structure parameters, *w* = 380 nm and *h* = 200 nm correspond to 660 nm and 970 nm, emission and absorption peaks, respectively, for the proposed material construction using Er^3+^/Yb^3+^ doped NaYF_4_ UC material [24]. The simulation assumed experimentally determined UC material and silicon refractive indexes [25,26], fitted to Drude-Lorentz and Drude-critical point models, respectively. Figure 2a confirms 660 nm and 970 nm resonance for the *w* = 380 nm, *h* = 200 nm, and *t* = 20 nm structure. We employed a freestanding UC film with thickness = 40 nm and the same volume as the UC slots on Si blocks as the reference structure. The reference film exhibited absorbance <1.2% over the investigated wavelength region, whereas the proposed UC slot structure exhibited strongly enhanced absorption ≈30% at 970 nm, even with the low filling factor = 0.0975 (ratio between UC slot area and total area).

Figure 2b,c shows that the resonance electric field profiles for 970 nm incident light exhibit strongly enhanced Ex polarized incident light in the UC slot array. Electric field intensity was strongly concentrated in the low index UC slot region rather than the high index Si block region, due to the slot waveguide mode, with maximum enhanced electric field intensity ≈30× incident wave intensity. Thus, the induced waveguide mode provides strong electric field intensity at lower index layers for vertically polarized incident light for the high-low-high index dielectric layer waveguide [27,28,29].

**Figure 3:**
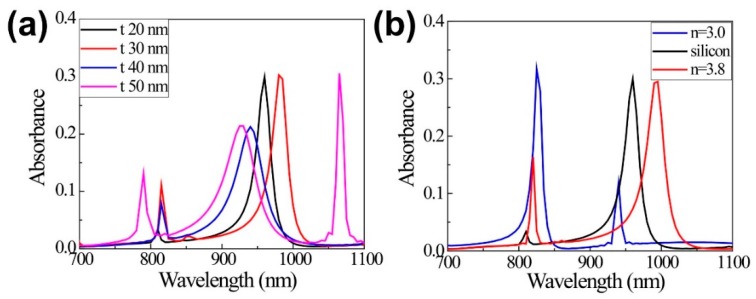
(**a**) Absorption spectra for different slot widths (*t*). Here, width of 380 nm and height of 200 nm are used. (**b**) Absorption spectra for different refractive indexes (n) of the block material.

Figure 3 shows the absorption dependencies on the UC slot width (*t*) and refractive index (n) of the block material. When the width (*t*) increases from 20 nm to 30 nm, 40 nm, and 50 nm, the absorption peak shifts from 970 nm to 983 nm, 940 nm, and 930 nm, showing the complicated wavelength shift behavior, a slight blue shift, and large red shifts in sequence (Figure 3a). This shift is attributed to the fact that the effective index of the slot mode becomes higher in the smaller width [27] and the confinement factor has a maximum value in a certain slot width [28]. The absorbance reaches 30% for the slots with the width of 20 nm and 30 nm and is approximately 20% for the slots with the width of 40 nm and 50 nm, respectively. However, considering the area-filling factors, 0.0975, 0.141, 0.181, and 0.219 of the UC material for the widths of 20 nm, 30 nm, 40 nm, and 50 nm, the slot with *t* = 20 nm shows the highest absorbance per unit UC volume. In the slot with a width of 50 nm, the other mode is also observed at a wavelength of 1065 nm.

On the other hand, as the refractive index of the block material increases from 3.0 (blue), and silicon (black), to 3.8 (red), the absorption peak shifts to the longer wavelength and the absorbance decrease slightly from 33% and 30%, to 28%. The optical dielectric function of the silicon includes the material dispersion depending on the wavelength.

## 3. Proposed Upconversion Slot Array Spontaneous Emission Enhancement

**Figure 4:**
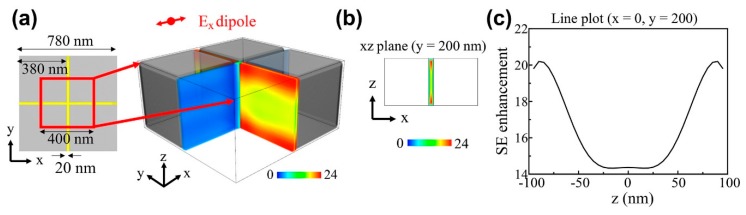
Spontaneous emission (SE) enhancement as functions of *Ex* dipole source location; (**b**) is SE enhancement in the XZ plane at *y* = 200 nm corresponding to (**a**); (**c**) is SE enhancement along the *z*-direction for (b).

We investigated converted visible light (660 nm) emission properties by calculating spontaneous emission (SE) for a dipole source excited in the UC slot array structure and reference UC film. The randomly polarized dipole SE emitters were represented by linear superposition of *Ex*, *Ey*, and *Ez* polarized dipole sources, and SE enhancement rates for horizontally polarized (*Ex*) or perpendicularly polarized (*Ez*) 660 nm dipole emitters were investigated. As expected, *Ey* polarized dipole emitter shows the same SE enhancement as the *Ex* polarized dipole source due to square lattice geometry. SE enhancement can be calculated as

(1) $$R_{\mathrm{SE}}=\frac{P_{\mathrm{UC}}}{P_{Bu\mathrm{lk}}},$$

where $P_{UC}$ and $P_{Bu\mathrm{lk}}$ are the dipole source emitted powers in the UC slot array and bulk UC material structure, respectively. We assume the bulk UC fills the entire space, and the corresponding SE is the natural decay rate. The total output power from the dipole source can be estimated by integrating the structure’s outgoing Poynting vectors. We scanned the position of the dipole source with 5 nm spatial resolution and set the calculation region to 1/8 of a unit cell to take advantage of the structure symmetry, as shown in Figure 4.

Figure 4a–c shows SE enhancement as a function of the *Ex* dipole source position. As *Ex* polarized incident light excites the slot waveguide mode along the *y*-direction UC slot, strong SE enhancement only occurs in the y direction (Figure 4a). SE in the *x*-direction slot is similar to that for bulk UC.

Figure 4b,c shows that SE is strongly enhanced at the UC slot vertical edges (Figure 4b, red), with maximum SE enhancement ≈24 × bulk UC, and average enhancement over slot = 10.36. The *Ey* dipole source has the same spatial SE dependence as *Ex*, aside from 90° rotation, whereas *Ez* dipole source emission is enhanced toward UC slot vertical center, with maximum and average enhancement = 2.7 and 1.9, respectively.

The SE enhancement rate of the cross-patterned UC slot array was enhanced 7.54-fold compared with bulk UC material considering *Ex*, *Ey*, and *Ex* dipole sources. In contrast, 40-mm-thick reference film provided 0.99 and 0.24 enhancements for *Ex* and *Ez* dipole sources, respectively, providing 0.74 average enhancement. Thus, the proposed UC slot construction provided 9.6 × the reference film structure enhancement. Table 1 compares UC slot array and reference film structure absorption and emission rates at 970 nm and 660 nm.

We estimated the total wavelength conversion efficiency enhancement as the combination of absorption (25) and SE (9.6) enhancement, i.e., total enhancement ≈240.

**Figure 5:**
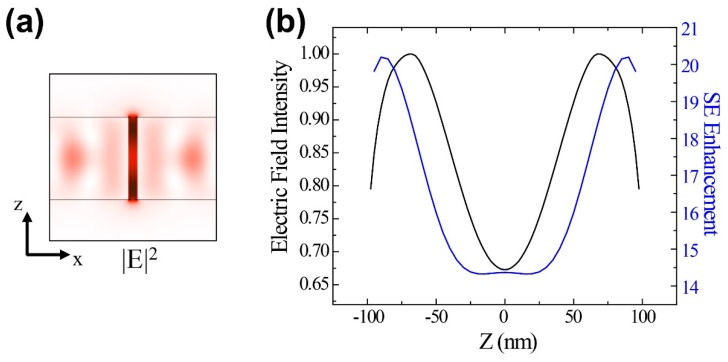
(**a**) Electric field intensity in the 660 nm resonance mode XZ plane (*y* = 200 nm). (**b**) Electric field intensity and spontaneous emission (SE) enhancement from Figure 3c along the *z*-direction at the UC slot center (*y* = 200 nm).

To identify the mechanism for the strong SE enhancement for *Ex* and *Ey* polarized dipole sources, we investigated the resonance mode excited in the UC slot array by placing a dipole source in the slot. Figure 5a shows the resultant electric field intensity mode profile in the XZ plane for *y* = 200 nm for the 660 nm mode.

A strong electric field occurs at the top and bottom of the UC slot (Figure 5a, dark red). SE enhancement along the *z*-direction in the UC slot (Figure 4c and Figure 5b, blue curve) can be explained by the Purcell effect due to the electric field intensity mode profile (Figure 5b, black curve). Since the Purcell effect is due to the electric field parallel to the dipole direction of an emitter, the similar spatial distributions for the resonant mode and SE enhancement confirm that the enhancement originates from the Purcell effect.

## 4. Conclusions

In this study, we designed a cross-patterned UC material slot array structure to enhance UC for practical solar cell and similar devices using Er^3+^/Yb^3+^ doped NaYF_4_ on silicon substrate. The proposed device achieved 25-fold enhanced incident light (970 nm) absorption and 9.6-fold SE enhancement of 660 nm emission compared with a reference 40-mm-thick uniform UC layer. These large enhancements originated from the two resonance slot modes targeted to match the UC material absorption and emission wavelengths. The double resonant modes provided overall 240-fold increased UC from the proposed cross-patterned UC slot array embedded in the silicon layer.

The proposed structure can be implemented by introducing UC material in the pre-defined air slots of the silicon layer using an atomic layer deposition [30] or growing amorphous silicon layer on the soft lithographically nanopatterned UC slot using the sol-gel method [31].

The enhanced wavelength conversion can be practically applied for photovoltaic devices based on surface plasmons and Mie resonances as well as slot mode, and the optical efficiency enhancement can be applied to various optical phenomena such as down conversion [32,33,34] and second harmonic generation [35,36,37].

## Figures and Tables

**Table 1:** Incident light (970 nm) absorption and spontaneous emission (SE, 660 nm) enhancement for the proposed upconversion (UC) slot array and 40-mm-thick reference UC film.

Process	Polarization	UC Slot Array	Reference UC Film	Multiplier
Absolute absorption (970 nm)		0.30	0.012	×25
SE enhancement (660 nm)	*Ex*	10.36	0.99	×10.5
	*Ey*	10.36	0.99	×10.5
	*Ez*	1.9	0.24	×7.9
Average SE enhancement		7.54	0.74	×9.6
Wavelength conversion efficiency				×242.5

## References

[1] Nyk M., Kumar R., Ohulchanskyy T.Y., Bergey E.J., Prasad P.N. (2008). High Contrast in Vitro and in Vivo Photoluminescence Bioimaging Using Near Infrared to Near Infrared Up-Conversion in Tm^3+^ and Yb^3+^ Doped Fluoride Nanophosphors. Nano Lett..

[2] Xiong L.-Q., Chen Z.-G., Yu M.-X., Li F.-Y., Liu C., Huang C.-H. (2009). Synthesis, characterization, and in vivo targeted imaging of amine-functionalized rare-earth up-converting nanophosphors. Biomaterials.

[3] Fang W., Wei Y. (2016). Upconversion nanoparticle as a theranostic agent for tumor imaging and therapy. J. Innov. Opt. Health Sci..

[4] van Sark W.G., de Wild J., Rath J.K., Meijerink A., Schropp R.E. (2013). Upconversion in solar cells. Nanoscale Res. Lett..

[5] Cheng Y.Y., Fückel B., MacQueen R.W., Khoury T., Clady R.G.C.R., Schulze T.F., Ekins-Daukes N.J., Crossley M.J., Stannowski B., Lips K. (2012). Improving the light-harvesting of amorphous silicon solar cells with photochemical upconversion. Energy Environ. Sci..

[6] Goldschmidt J.C., Fischer S. (2015). Upconversion for photovoltaics—A review of materials, devices and concepts for performance enhancement. Adv. Opt. Mater..

[7] Lee K.-T., Park J.-H., Kwon S.J., Kwon H.-K., Kyhm J., Kwak K.-W., Jang H.S., Kim S.Y., Han J.S., Lee S.-H. (2015). Simultaneous Enhancement of Upconversion and Downshifting Luminescence via Plasmonic Structure. Nano Lett..

[8] Schulze T.F., Schmidt T.W. (2015). Photochemical upconversion: Present status and prospects for its application to solar energy conversion. Energy Environ. Sci..

[9] Andrews D.L., Curutchet C., Scholes G.D. (2011). Resonance energy transfer: Beyond the limits. Laser Photon. Rev..

[10] Shalav A., Richards B.S., Trupke T., Krämer K.W., Güdel H.U. (2005). Application of NaYF4:Er3+ up-converting phosphors for enhanced near-infrared silicon solar cell response. Appl. Phys. Lett..

[11] Zhao C., Kong X., Liu X., Tu L., Wu F., Zhang Y., Liu K., Zeng Q., Zhang H. (2013). Li+ ion doping: An approach for improving the crystallinity and upconversion emissions of NaYF4:Yb^3+^, Tm^3+^ nanoparticles. Nanoscale.

[12] Sun Q.-C., Mundoor H., Ribot J.C., Singh V., Smalyukh I.I., Nagpal P. (2014). Plasmon-Enhanced Energy Transfer for Improved Upconversion of Infrared Radiation in Doped-Lanthanide Nanocrystals. Nano Lett..

[13] Lee G.Y., Jung K., Jang H.S., Kyhm J., Han I.K., Park B., Ju H., Kwon S.J., Ko H. (2016). Upconversion luminescence enhancement in plasmonic architecture with random assembly of metal nanodomes. Nanoscale.

[14] Park K., Jung K., Kwon S.J., Jang H.S., Byun D., Han I.K., Ko H. (2016). Plasmonic Nanowire-Enhanced Upconversion Luminescence for Anticounterfeit Devices. Adv. Funct. Mater..

[15] Purcell E.M., Torrey H.C., Pound R.V. (1946). Resonance absorption by nuclear magnetic moments in a solid. Phys. Rev..

[16] Gérard J.M., Gayral B. (1999). Strong Purcell effect for InAs quantum boxes in three-dimensional solid-state microcavities. J. Lightw. Technol..

[17] Lee Y.J., Moon K., Kwon S.-H. (2019). Enhanced Conversion Process in a Sub-wavelength Thin Upconversion Layer by Using Metamaterial Mirror. Plasmonics.

[18] Moon K., Lee Y., Hong S., Kwon S.-H., Moon K., Lee Y.J., Hong S., Kwon S.-H. (2017). Wavelength Conversion Enhancement Achieved by Using Resonance in an Array of Nanocylinders. Appl. Sci..

[19] Saboktakin M., Ye X., Chettiar U.K., Engheta N., Murray C.B., Kagan C.R. (2013). Plasmonic Enhancement of Nanophosphor Upconversion Luminescence in Au Nanohole Arrays. ACS Nano.

[20] Aouani H., Rahmani M., Navarro-Cía M., Maier S.A. (2014). Third-harmonic-upconversion enhancement from a single semiconductor nanoparticle coupled to a plasmonic antenna. Nat. Nanotechnol..

[21] Melik-Gaykazyan E.V., Shcherbakov M.R., Shorokhov A.S., Staude I., Brener I., Neshev D.N., Kivshar Y.S., Fedyanin A.A. (2017). Third-harmonic generation from Mie-type resonances of isolated all-dielectric nanoparticles. Philos. Trans. R. Soc. A Math. Phys. Eng. Sci..

[22] Fischer S., Goldschmidt J.C., Löper P., Bauer G.H., Brüggemann R., Krämer K., Biner D., Hermle M., Glunz S.W. (2010). Enhancement of silicon solar cell efficiency by upconversion: Optical and electrical characterization. J. Appl. Phys..

[23] de Wild J., Meijerink A., Rath J.K., van Sark W.G.J.H.M., Schropp R.E.I. (2010). Towards upconversion for amorphous silicon solar cells. Sol. Energy Mater. Sol. Cells.

[24] Haase M., Schäfer H. (2011). Upconverting Nanoparticles. Angew. Chem. Int. Ed..

[25] Sokolov V.I., Zvyagin A.V., Igumnov S.M., Molchanova S.I., Nazarov M.M., Nechaev A.V., Savelyev A.G., Tyutyunov A.A., Khaydukov E.V., Panchenko V.Y. (2015). Determination of the refractive index of β-NaYF4/Yb3+/Er3+/Tm3+ nanocrystals using spectroscopic refractometry. Opt. Spectrosc..

[26] Schinke C., Christian Peest P., Schmidt J., Brendel R., Bothe K., Vogt M.R., Kröger I., Winter S., Schirmacher A., Lim S. (2015). Uncertainty analysis for the coefficient of band-to-band absorption of crystalline silicon. AIP Adv..

[27] Xu Q., Almeida V.R., Panepucci R.R., Lipson M. (2004). Experimental demonstration of guiding and confining light in nanometer-size low-refractive-index material. Opt. Lett..

[28] Feng N.-N., Michel J., Kimerling L.C. (2006). Optical Field Concentration in Low-Index Waveguides. IEEE J. Quantum Electron..

[29] Anderson P.A., Schmidt B.S., Lipson M. (2006). High confinement in silicon slot waveguides with sharp bends. Opt. Express.

[30] Giedraityte Z., Tuomisto M., Lastusaari M., Karppinen M. (2018). Three- and Two-Photon NIR-to-Vis (Yb,Er) Upconversion from ALD/MLD-Fabricated Molecular Hybrid Thin Films. ACS Appl. Mater. Interfaces.

[31] Park H., Yoo G.Y., Kim M.-S., Kim K., Lee C., Park S., Kim W. (2017). Thin film fabrication of upconversion lanthanide-doped NaYF4 by a sol-gel method and soft lithographical nanopatterning. J. Alloys Compd..

[32] Wegh R., Donker H., van Loef E.V., Oskam K., Meijerink A. (2000). Quantum cutting through downconversion in rare-earth compounds. J. Lumin..

[33] Oskam K., Wegh R., Donker H., van Loef E.V., Meijerink A. (2000). Downconversion: A new route to visible quantum cutting. J. Alloys Compd..

[34] Richards B.S. (2006). Luminescent layers for enhanced silicon solar cell performance: Down-conversion. Sol. Energy Mater. Sol. Cells.

[35] Kozlovsky W.J., Nabors C.D., Byer R.L. (1988). Efficient second harmonic generation of a diode-laser-pumped CW Nd:YAG laser using monolithic MgO:LiNbO/sub 3/external resonant cavities. IEEE J. Quantum Electron..

[36] Thyagarajan K., Rivier S., Lovera A., Martin O.J.F. (2012). Enhanced second-harmonic generation from double resonant plasmonic antennae. Opt. Express.

[37] Celebrano M., Wu X., Baselli M., Großmann S., Biagioni P., Locatelli A., De Angelis C., Cerullo G., Osellame R., Hecht B. (2015). Mode matching in multiresonant plasmonic nanoantennas for enhanced second harmonic generation. Nat. Nanotechnol..

